# Approach and Withdrawal Motivation in Schizophrenia: An Examination of Frontal Brain Asymmetric Activity

**DOI:** 10.1371/journal.pone.0110007

**Published:** 2014-10-13

**Authors:** William P. Horan, Jonathan K. Wynn, Ian Mathis, Gregory A. Miller, Michael F. Green

**Affiliations:** 1 VA Greater Los Angeles Healthcare System, Los Angeles, California, United States of America; 2 University of California Los Angeles, Los Angeles, California, United States of America; Laureate Institute for Brain Research and The University of Oklahoma, United States of America

## Abstract

Although motivational disturbances are common in schizophrenia, their neurophysiological and psychological basis is poorly understood. This electroencephalography (EEG) study examined the well-established motivational direction model of asymmetric frontal brain activity in schizophrenia. According to this model, relative left frontal activity in the resting EEG reflects enhanced approach motivation tendencies, whereas relative right frontal activity reflects enhanced withdrawal motivation tendencies. Twenty-five schizophrenia outpatients and 25 healthy controls completed resting EEG assessments of frontal asymmetry in the alpha frequency band (8–12 Hz), as well as a self-report measure of behavioral activation and inhibition system (BIS/BAS) sensitivity. Patients showed an atypical pattern of differences from controls. On the EEG measure patients failed to show the left lateralized activity that was present in controls, suggesting diminished approach motivation. On the self-report measure, patients reported higher BIS sensitivity than controls, which is typically interpreted as heightened withdrawal motivation. EEG asymmetry scores did not significantly correlate with BIS/BAS scores or with clinical symptom ratings among patients. The overall pattern suggests a motivational disturbance in schizophrenia characterized by elements of both diminished approach and elevated withdrawal tendencies.

## Introduction

Motivational impairment has been linked to schizophrenia since Kraepelin’s [1] early descriptions of a “weakening of the wellsprings of volition” in this disorder. Motivational disturbances figure prominently in contemporary descriptions of schizophrenia, particularly of negative symptoms such as anhedonia, avolition, and asociality, which are strongly associated with poor treatment outcome and functional impairment [2]. Despite the theoretical and functional significance of motivational disturbances in schizophrenia, little is known about their neurophysiological and psychological bases [3], [4]. The current study addresses this issue by examining an influential model of approach and withdrawal motivational systems assessed with electrophysiology (EEG) and self-report measures in schizophrenia.

Considerable evidence supports the motivational direction model of asymmetric frontal brain activity [5]–[10]. According to this model, two fundamental and distinct motivational systems, one supporting approach behaviors and another supporting withdrawal behaviors, are associated with separate neural circuits that involve different regions of the frontal cortex. The approach motivational system is thought to modulate planning for and reactions to appetitive or rewarding stimuli associated with emotions (e.g., enthusiasm, pleasure, anger) and behaviors that prompt us approach desired goals and rewards. The avoidance motivational system, in contrast, is thought to modulate processing of aversive stimuli and to generate corresponding withdrawal/avoidance (e.g., fear, anxiety) emotional and behavioral responses to avert threats and punishment. The neural correlates of these systems have primarily been examined through resting EEG assessments of lateralized frontal alpha band (8–12 Hz) power, which traditionally has been interpreted as *inversely* related to regional activity. Left frontal activity (i.e., lower left alpha power) is believed to reflect approach motivation, whereas right frontal activity (i.e., lower right alpha power) is associated with withdrawal motivation. These asymmetrical frontal EEG findings are supported by lesion studies, though hemodynamic studies have been less consistent [9],[11]–[13].

Frontal asymmetries assessed in the resting EEG are believed to relate to stable dispositional motivational tendencies. Consistent with this notion, several studies demonstrate associations between degree of lateralization of activation and scores on conceptually related self-report motivational trait measures. For example, in studies using the BIS/BAS scales [14], which were designed to assess sensitivities of the behavioral inhibition and activation systems as originally described by Gray [15], higher BAS scores correlate with higher relative left frontal activity, and higher BIS scores often, though not always, correlate with higher relative right frontal activity [7]. A large number of studies also demonstrate that frontal asymmetries are related to various clinical conditions [8], [16]. For example, elevated right frontal lateralization is associated with certain types of anxiety disorders, reduced left lateralization is associated with vulnerability to anhedonic depression, and elevated left is associated with vulnerability to mania [16], [17]. Hemodynamic neuroimaging studies have provided support for some of the EEG findings [18]–[20].

Frontal asymmetry and motivational tendencies have been examined explicitly in schizophrenia in only two prior studies. One found that recent-onset patients showed more right frontal lateralization (using a single unconventional pair of electrodes) than healthy controls during a two-minute recording with eyes closed [21], a somewhat shorter epoch than recommended [22], [23]. The other examined chronically ill outpatients with schizophrenia spectrum disorders (no comparison group) and found that asymmetry scores showed good stability over 36 months and that leftward lateralization correlated with higher positive symptoms [24], [25]. It is also worth noting that two studies compared schizophrenia and healthy control groups on the BIS/BAS scales without EEG; both found that patients reported significantly elevated BIS scores, which is consistent with greater right frontal lateralization, but not differences in BAS scores [26], [27].

The current study assessed both resting frontal EEG asymmetries and self-reported approach/withdrawal tendencies in schizophrenia. The prior literature, though very limited, led to the prediction that patients would show relatively more right frontal lateralization and higher BIS scores than matched healthy controls. We also explored whether frontal asymmetries relate to individual differences on the BIS/BAS and to symptom levels among patients.

## Methods

### Participants

Twenty-five outpatients with schizophrenia and 25 healthy control subjects participated in this research. Schizophrenia patients were recruited from outpatient treatment clinics at the Veterans Affairs (VA) Greater Los Angeles Healthcare System and through presentations in the community. Patients met criteria for schizophrenia based on the Structured Clinical Interview for DSM-IV Axis I Disorders (SCID [28]). None of the patients was in a major depressive episode at the time of testing. Additional exclusion criteria for patients included: substance abuse or dependence in the last six months; IQ<70 based on chart reviews; a history of loss of consciousness for more than one hour; an identifiable neurological disorder; or insufficient fluency in English. All patients were clinically stable as defined by: no hospitalizations in the past 3 months, no changes in living situation in the past 2 months, and no medication changes in the past 6 weeks. All patients were at clinically determined dosages, with 19 receiving atypical antipsychotic medications, 2 receiving typical antipsychotic medications, and 4 receiving both types of medication.

Healthy controls were recruited through flyers posted in local newspapers, websites, and posted advertisements. An initial screening interview excluded potential control participants with identifiable neurological disorder or head injury, had schizophrenia or other psychotic disorder in a first-degree relative, or were not sufficiently fluent in English. Potential controls were screened with the SCID and excluded for history of schizophrenia or other psychotic disorder, bipolar disorder, recurrent depression, lifetime history of substance dependence, or any substance abuse in the last 6 months. Potential controls were also administered portions of the Structured Clinical Interview for DSM-IV Axis II Disorders (SCID-II [29]) and excluded if they had avoidant, paranoid, schizoid, or schizotypal personality disorder.

All SCID interviewers were trained through the Treatment Unit of the Department of Veterans Affairs VISN 22 Mental Illness Research, Education, and Clinical Center (MIRECC) to a minimum kappa of 0.75 for key psychotic and mood items [30]. All participants had the capacity to give informed consent and provided written informed consent after all procedures were fully explained in accordance with procedures approved by the Institutional Review Boards at UCLA and the VA Greater Los Angeles Healthcare System.

### Symptom ratings

#### BPRS

For all patients, psychiatric symptoms during the previous month were rated using the expanded 24-item UCLA version of the Brief Psychiatric Rating Scale (BPRS [31], [32]) by a trained rater [33]. Ratings from the positive and negative symptom subscales, as well as total scores, were examined [34].

### Self-report measure

#### BIS/BAS scales

All participants completed the BIS/BAS [14], a 20-item instrument that assesses dispositional sensitivity of the avoidance motivational system and the approach motivational system. Participants rate each item on a 1 (strongly agree) to 4 (strongly disagree) scale. Sample BIS items include “I worry about making mistakes”, “Criticism or scolding hurts me quite a bit.” Sample BAS items include “I crave excitement and new sensations,” “I go out of my way to get the things I want.” The BAS is composed of three subscales reflecting aspects of incentive responsiveness: Drive, Fun Seeking, and Reward Responsiveness. Items on each scale are summed. Two patient participants did not complete the BIS/BAS.

### EEG data acquisition and analysis

The research design and methods reflect standard procedures for studies of frontal EEG asymmetry [35]. Participants had their resting EEG recorded for four one-minute segments with eyes open and four one-minute segments with eyes closed, which was administered in one of two counterbalanced orders. EEG activity was collected using a 64-channel Neuroscan SynAmps2 amplifier and a Neuroscan 64-channel QuickCap (Compumedics USA, Charlotte, NC). It took approximately 20 minutes to put on the cap and check the electrodes. Data were sampled at 500 Hz with filter settings of 0 to 100 Hz in DC acquisition mode. 64 cap-mounted, equidistant, sintered Ag-AgCl electrodes were positioned in the QuickCap using the 10–10 international placement system. Additionally, four electrodes were used to measure bipolar horizontal electrooculogram (EOG; placed on the outer canthus of the left and right eye) and bipolar vertical EOG (placed above and below the left eye). All electrodes were referenced to a point halfway between electrodes Cz and CPz, and a forehead ground was employed. Subjects were seated in a comfortable chair and the lights were dimmed during recording. Assessments were conducted between 10am–3pm. No relaxation procedures were used before measuring EEG.

Off-line analysis was performed using Brain Vision Analyzer software (Brain Products, Munich, Germany). All EEG data were re-referenced to the average of the mastoids [36] (All analyses were re-run with re-referencing to the average of all electrodes; results were essentially identical) and band-pass filtered using a zero phase shift Butterworth filter with cutoffs of 1–100 Hz (24 dB/octave rolloff), along with a 60 Hz notch filter. EEG was corrected for blinks and eye movements using the method developed by Gratton and colleagues [37], [38]. Each condition (eyes open, eyes closed) was then divided into 240 overlapping epochs of 1.024 s duration. Epochs that exceeded +/−100 µV at specific channels (F1, F2, F3, F4, F5, F6, F7, F8; P3, P4) were rejected using a semi-automated procedure. Artifact-free epochs were analyzed with a fast Fourier transform (FFT), using a Hamming window of 75% and padding of the epoch with zeroes to 2.048 seconds, resulting in a nominal resolution of 0.49 Hz. Epochs were then averaged and power within the alpha (8–13 Hz) frequency band was calculated. Primary analyses focused on electrodes F4 & F3, which are the most commonly assessed sites in the frontal asymmetry literature [7], [16]. We also examined electrodes F8 & F7 as these are sometimes included in frontal asymmetry investigations, and the parietal sites P4 & P3 as these are sometimes used in non-frontal control analyses or the context of other models of laterality [39]. Asymmetry scores were calculated by subtracting the natural log-transformed scores (i.e., ln[Right] – ln[Left]) for each homologous left and right pair. Because alpha power is often interpreted as inversely related to cortical activity, higher values on this index reflect greater left activity [35].

### Data Analyses

For demographic data, group differences for continuous variables were evaluated with *t*-tests and for categorical variables with chi-square tests; descriptive data for clinical symptom ratings in the patient group are also presented. For the main data analyses, group differences in alpha asymmetry scores and self-reported BIS/BAS scores were evaluated with independent samples t-tests. Finally, we evaluated whether asymmetry scores were associated with BIS/BAS scores and symptoms (patients only) using Pearson correlation coefficients within each group.

## Results

### Demographic and clinical characteristics

Demographic information for both groups and clinical data for the schizophrenia group are presented in Table 1. The groups did not significantly differ in sex or ethnicity. Although the patients had lower personal education levels than controls, the groups did not differ in parental education. There was a significant age difference between the groups; patients were older than controls. To evaluate the potential impact of age on the results, we computed correlations between age and all of EEG, self-report, and symptom (for patients) variables separately within each group. There were no significant correlations involving age and we therefore did not account for age in the primary data analyses. There were also no significant correlations between CPZ equivalents and any other study measure.

**Table 1:** Demographic and Clinical Data.

	Schizophrenia	Controls	Statistic
	(N = 25)	(N = 25)	
Sex (% male)	76.0%	68.0%	X^2^ (1,50) = .40
Age (SD)	49.3 (7.7)	43.6 (9.43)	t(48) = 2.33*
Ethnicity			X^2^ (4,50) = 3.71
White	33.3%	52.0%	
African American	41.7%	24.0%	
Asian	8.3%	12.0%	
Hispanic	12.5%	4.0%	
Other	4.2%	8.0%	
Marital status			X^2^ (2,50) = 3.80
Never married	60.0.%	60.0%	
Currently married	4.0%	20.0%	
Ever married	36.0%	20.0%	
Education (SD)	13.0 (1.5)	15.0 (1.4)	t(56) = –4.97**
Parental education (SD)	14.0 (3.5)	15.2 (2.6)	t(56) = –1.31
Age of onset (SD)	21.7 (5.3)		
Duration of illness (SD)	27.3 (8.0)		
CPZ equivalents (SD)	326.36 (205.94)		
BPRS			
Positive symptoms (SD)	1.9 (0.7)		
Depression (SD)	2.0 (0.7)		
Negative symptoms (SD)	1.8 (0.7)		
Agitation (SD)	1.1 (0.1)		
Total (SD)	40.8 (8.1)		

Notes: BPRS = Brief Psychiatric Rating Scale;

*p<.05;

**p<.001.

The schizophrenia group had a typical age of onset and was chronically ill. They showed mild to moderate levels of clinical symptoms at the time of testing that are comparable to prior studies of stabilized outpatients [40], [41].

### Group comparisons on alpha asymmetry

Descriptive statistics and results of between-group comparisons are summarized in Table 2. For the primary measure of frontal asymmetry, F4–F3, patients had negative scores that were significantly lower than those of controls. This pattern suggests patients showed either more right lateralization or less left lateralization than controls. To further characterize this between-group difference, we conducted a Group X Hemisphere ANOVA, which revealed a significant interaction effect, F(1,48) = 4.56, p<.05, but non-significant effects for group and hemisphere (p’s>.45). Follow up t-tests indicated that controls showed greater left than right activation, t(24) = 2.47, p<.05, whereas patients did not show a significant difference between left and right activation, t(24) = –1.02, p>.30); there were no between-group differences within either the left or right hemisphere (p’s>.25). Thus, patients failed to show the left lateralization that was present in the control group.

**Table 2:** Group Differences on Asymmetry Scores.

	Schizophrenia	Controls	t
	(N = 25)	(N = 25)	
F4 minus F3	−.18	.26	−2.14*
	(.88)	(.52)	
F8 minus F7	−.47	−.02	−1.56
	(1.07)	(.95)	
P4 minus P3	.32	−.02	1.06
	(.96)	(1.29)	

Notes: Positive values correspond to greater relative left activity and negative values correspond to greater relative right activity.

*p<.05.

Although patients had numerically lower mean scores on the secondary index (F8–F7) than controls, the groups did not significantly differ. Finally, for the analysis of the P4–P3 electrodes, there was no significant group difference in parietal asymmetry scores.

### Group comparisons on self-report motivation

As shown in Table 3, patients reported significantly higher BIS scores than controls, indicating that patients reported greater behavioral inhibition sensitivity. There were no significant group differences on the BAS total scores or on any of the subscale scores. Internal consistency estimates for each subscale were acceptable within the patient and control groups.

**Table 3:** Between-Group Comparisons on the BIS/BAS.

	Schizophrenia	Controls	t
	(N = 23)	(N = 25)	
BIS Total	21.35 (3.54)	17.92 (4.11)	3.08*
	**α** = .63	**α** = .83	
BAS Total	39.74 (7.89)	39.76 (4.51)	−0.01
	**α** = .90	**α** = .77	
BAS Drive	11.04 (3.31)	10.84 (1.93)	0.26
	**α** = .87	**α** = .60	
BAS Fun	11.61 (2.76)	11.60 (2.40)	0.01
	**α** = .68	**α** = .74	
BAS Reward	17.09 (2.76)	17.32 (2.23)	−0.32
	**α** = .78	**α** = .69	

Notes: BIS = Behavioral Inhibition System scale; BAS = Behavioral Activation System scale; Standard deviations appear in parentheses. **α** = Chronbach’s coefficient alpha. *p<.005.

### Correlations with self-reported motivation and symptoms

Among patients the F4–F3 asymmetry index was not significantly correlated with BIS, BAS, or any of the three BAS subscales (all r’s<.17, p’s>.40). Similarly, for controls, the F3–F4 asymmetry index was not significantly correlated with BIS, BAS, or BAS subscale scores (all r’s<.19, p’s>.38). There were no significant correlations between asymmetry and total symptoms or symptom subscale scores on the BPRS (all r’s<.30, p’s>.15). Results for the correlation analyses were essentially identical using Spearman correlation coefficients.

## Conclusions

In this first study to evaluate approach and withdrawal motivation in schizophrenia using both self-report and EEG measures, patients showed an atypical pattern of differences from controls. On the resting EEG measure patients failed to show the left lateralized activity that was present in controls, suggesting diminished approach motivation in the schizophrenia group. On the self-report measure, patients reported higher BIS sensitivity than controls, which is typically interpreted as heightened withdrawal motivation. EEG asymmetry scores however, were not significantly correlated with self-reported motivational traits in either group. The overall pattern of group differences converges to suggest a motivational disturbance in schizophrenia characterized by elements of both diminished approach tendencies and elevated withdrawal tendencies.

The current between-group motivational differences confirm and extend the few prior studies using either EEG or self-report measure alone. Regarding EEG, the two groups showed opposite asymmetry patterns at mid-frontal sites, the most commonly examined sites in the frontal asymmetry literature [7], [16], with a left bias in controls versus a right bias in patients. This finding is broadly similar with an earlier report of right frontal lateralization in recent-onset schizophrenia patients [21]. The current study had several methodological strengths compared to the earlier report, including data from a much longer recording epoch, analyses on a commonly-examined set of frontal electrode sites, and well-established data analysis procedures. It also extends the earlier report by demonstrating that the patients’ abnormal asymmetry score appears to be more a reflection of diminished left lateralization rather than greater right lateralization. Diminished left lateralization is strongly associated with depression, particularly anhedonic depression, as well as vulnerability to depression, and is thought to reflect reduced approach motivation and reward sensitivity [9], [10], [16]. A similar motivational disturbance is believed to underlie certain negative symptoms of schizophrenia, such as anhedonia and amotivation, though asymmetry scores were not associated with negative symptoms in the current study.

The patients’ elevated self-reported scores on the BIS scale, but normal scores on the BAS scales, also converge with the few prior relevant studies in schizophrenia [26], [27]. Elevated BIS scores reflect hyperactivity of the withdrawal motivational system and are associated with heightened sensitivity to threat, physiological arousal, and experience of negative emotions such as fear and distress [5]–[8], [10]. This pattern fits well with several lines of research in schizophrenia indicating that patients frequently show elevated trait negative affectivity, elevated reactivity to negative stimuli/stress and punishment feedback, and difficulty regulating negative emotions [42]–[44]. The heightened self-reported BIS (but normal BAS) sensitivity seen in schizophrenia patients is comparable to the pattern seen in certain anxiety disorders, such as social anxiety and panic disorders [9], [10], [16].

Overall, the current findings suggest that when it comes to understanding motivational factors that hold people with schizophrenia back from pursuing personally relevant goals, it is important to consider multiple processes. Diminished initiation and persistence in goal-directed activities may stem from a lack of desire to pursue potentially rewarding outcomes and/or a strong drive to avoid potentially unpleasant emotions and cognitions associated with efforts to pursue desired outcomes.

Results from the current study are not fully consistent with prior studies, in that frontal asymmetry scores did not significantly correlate with symptom levels or with BIS/BAS scores. Regarding symptoms, Jetha et al. [24] found that left frontal lateralization correlated with positive symptoms in a sample with a large proportion of patients with prominent positive symptoms. The lack of symptom correlates in the present stabilized outpatient sample, therefore, suggests that frontal asymmetry scores are not simply a marker of clinical state. As noted above, it was somewhat unexpected that negative symptoms were not correlated with asymmetry as they are conceptually related to diminished BAS activity. This may reflect the use of the BPRS negative symptom scale, which focuses on expressive negative symptoms rather than motivation- and pleasure-related negative symptoms [2].

We also did not find significant correlations between frontal asymmetry and scores on the BIS/BAS scales in either patients or controls. Such correlations have been reported in some studies in clinical and healthy samples, though a number of studies have failed to find significant correlations, particularly for the BIS scale [7]. This absence of significant correlations for the BIS may partly reflect an emerging view that the withdrawal construct associated with rightward lateralization has considerably less overlap with the inhibition construct assessed by the BIS scale than originally suggested [7], [45], [46]. The extent to which these are overlapping versus distinct constructs is currently an active area of research. For example, BIS may correlate differentially with areas of dorsolateral prefrontal cortex that relate specifically to selecting approach goals, selecting withdrawal goals, and selecting goal-pursuit strategies [47]. Another factor that may account for this discrepancy is that resting EEG may not be as powerful to detect individual differences in motivational tendencies as task-activated EEG and fMRI paradigms [48], [49]. Future studies of schizophrenia would benefit from using approach- and withdrawal–related emotional challenge paradigms to obtain a potentially more sensitive index of the capacity to activate these motivational systems.

The current finding of increased withdrawal motivational tendencies in schizophrenia should be interpreted in the context of some limitations. First, this cross-sectional study of chronically ill patients cannot address the question of whether this pattern precedes the onset of schizophrenia or is the consequences of living with a severe mental illness. Although prior research demonstrating the same asymmetry pattern is present in recent-onset patients and that asymmetry scores show good longitudinal stability in schizophrenia is consistent with the notion that elevated right frontal lateralization reflects an enduring trait that is present from at least the early post-onset period [21], [25], further research is needed to address this issue (e.g., in high-risk subjects). A second limitation was reliance on the BIS/BAS as the sole measure of approach and withdrawal motivation. Spielberg et al. ([50], [51] also see [52]) developed a composite measure of approach/withdrawal motivation with better psychometric properties in nonclinical samples than the BIS alone. Third, resting frontal EEG asymmetry can be affected by current emotional state and other factors (e.g., fatigue, hunger) [49] that were not directly assessed in this study. Fourth, the patients were taking medications at clinically determined dosages and the impact on the current findings is unclear. Although CPZ equivalents were not significantly correlated with EEG or self-report measures, and prior research has not found that psychotropic medications impact resting frontal asymmetry [16], research in unmedicated patients is required to directly address this question. Fifth, as mentioned above, we were not able to directly measure the relationship between frontal asymmetry clinically rated motivational deficits since the BPRS assesses the expressive, but not the experiential, component of negative symptoms. Sixth, the samples were not matched for age, although supplementary analyses indicated that age did not relate to the study variables.

Although the approach and withdrawal motivational framework has been extensively studied in internalizing and externalizing disorders, the current findings suggest it may be informative for understanding motivational disturbances in the psychosis dimension of psychopathology as well. The framework has treatment implications for schizophrenia patients with elevated withdrawal motivation. For example, CBT, emotion regulation, and mindfulness interventions that target symptoms such as anxiety and hyper-arousal may be useful for these individuals, and frontal asymmetries have been found to provide an informative biomarker in the context of these types of treatment studies [53]–[55]. The general approach/withdrawal framework has also been applied to more specific investigations of functioning in the social domain [56]. Further investigation of social approach and withdrawal may be useful for isolating factors that contribute to the social difficulties that many individuals with schizophrenia experience.

## Supporting Information

Data S1 (SAV)
